# Postural control in early stages of Duchenne muscular dystrophy

**DOI:** 10.1590/1984-0462/2025/43/2024243

**Published:** 2025-06-27

**Authors:** Cristhina Bonilha Huster Siegle, Flaviana Kelly de Lima Maciel, Pedro Claudio Gonsales de Castro, Daniel Seiei Uehara Tamashiro, Cristina dos Santos Cardoso de Sá

**Affiliations:** a Universidade Federal de São Paulo – Santos (SP), Brazil.; b Faculdade de Medicina da Universidade de São Paulo – São Paulo (SP), Brazil.; c Escola Superior de Saúde do Alcoitão – Alcabideche, Portugal.

**Keywords:** Balance, Postural control, Evaluation study, Duchenne muscular dystrophy, Children, Equilíbrio, Controle postural, Estudo de avaliação, Distrofia muscular de Duchenne, Criança

## Abstract

**Objective:**

To verify whether there is an increase in the center of pressure (COP) displacement velocity and area in individuals with Duchenne muscular dystrophy (DMD) in Vignos stages 2 and 3, assessed under different sensory conditions; to verify whether COP displacement velocity and area increase within the same Vignos stage with changes in sensory conditions during postural control assessment.

**Methods:**

A cross-sectional study was conducted with individuals with DMD in Vignos stages 2 and 3, aged between 7 and 19 years old (mean 9.58, SD±3.22). Postural control was assessed using the Wii Balance Board and a modified sensory organization test. COP displacement velocity and area were recorded. Independent samples t-tests were performed to compare the average values of these variables between Vignos 2 and 3 under each sensory condition and within the same Vignos stage across different sensory conditions.

**Results:**

No significant difference were found in any analyzed variable between Vignos 2 and 3. However. A significant difference was observed when comparing sensory condition 1 with condition 4, within the same disease stage.

**Conclusions:**

Despite the decrease in muscle strength from Vignos 2 to 3, participants were able to employ motor strategies to maintain postural control. However, postural control performance significantly worsens with changes in sensory conditions within the same Vignos stage.

## RESUMO

**Objetivo:**

Verificar se há um aumento na velocidade e na área de deslocamento do centro de pressão (COP) em indivíduos com distrofia muscular de Duchenne (DMD) nos estágios Vignos 2 e 3, avaliados em diferentes condições sensoriais; além disso, verificar se há um aumento na velocidade e na área de deslocamento do COP nos participantes pertencentes ao mesmo Vignos, com mudanças nas condições sensoriais da avaliação de controle postural.

**Métodos:**

Estudo transversal realizado com indivíduos com DMD nos estágios Vignos 2 e 3, com idade entre 7 e 19 anos (média 9.58, DP±3.22). A plataforma do Nintendo Wii foi utilizada para a avaliação do controle postural, por meio do teste de organização sensorial modificado. A velocidade e área de deslocamento do COP foram registradas. O teste t para amostras independes foi realizado para comparar as médias das variáveis entre os estágios Vignos 2 e 3 em cada condição sensorial, bem como entre diferentes condições sensoriais dentro do mesmo Vignos.

**Resultados:**

Não foram encontradas diferenças significativas nas variáveis analisadas entre Vignos 2 e 3. No entanto, houve diferença significativa ao comparar os resultados das variáveis na condição 1 com a condição 4 dentro do mesmo estadiamento da doença.

**Conclusões:**

Apesar da diminuição da força muscular na progressão do estágio Vignos 2 para o 3, os participantes ainda apresentaram estratégias motoras para manter o mesmo controle postural. No entanto, observou-se uma piora significativa no controle postural com a variação das condições sensoriais em indivíduos de um mesmo Vignos.

## INTRODUCTION

 Postural control is essential for performing functional movements and skills such as sitting, standing, reaching, and activities of daily living.^1^ It is maintained through constant body oscillations, which generate small mediolateral and anteroposterior movements,^2,3^ and can be measured by posturography. As a gold standard measure for assessing postural control, the acceleration of the center of mass and displacement of the body’s center of pressure (COP) are analyzed using a force platform.^1^ Visual, vestibular, and somatosensory systems contribute to regulating this control.^2^ By altering the stiffness condition of the base of support and visual information, the influence of sensory components on postural control can be investigated.^2,4^

 In Duchenne muscular dystrophy (DMD), the muscles of the lower limbs and trunk are progressively affected over the course of the disease, compromising orthostatic posture maintenance. Balance deficits become apparent, along with falls, difficulty sitting and standing, Gowers’ sign, and an equine gait.^5^ Weakness of the trunk muscles generates an imbalance in this region, which may be related to the postural abnormalities observed in these individuals and the compensatory mechanisms that help maintain standing and sitting postures.^6^ Over time, these trunk deficits result in spinal deformities such as scoliosis and hyperlordosis, further affecting postural control, balance, and functional independence.^6^ In addition, the progression of muscle weakness in the lower limbs leads to compensatory mechanisms such as knee hyperextension and hip joint stiffness, which can further impair postural control and orthostatic posture maintenance.^7^

 A cross-sectional study found that individuals with DMD exhibit greater anteroposterior and mediolateral sway compared to a control group without DMD, with this characteristic already present in individuals aged five to six years.^3^ The same study suggested that early changes in postural control may predispose individuals to severe scoliosis in later stages.^3^ Another study, which used the modified sensory organization test (MSOT), did not find significant differences in COP excursion area but reported an increase in COP displacement velocity under the assessed conditions.^2^ To date, no studies have analyzed changes in postural control across different disease stages in individuals with DMD. Identifying these population characteristics could provide a basis for therapeutic interventions aimed at minimizing or delaying declines in postural control. 

 The primary aim of this study was to determine whether there is an increase in COP displacement velocity and displacement area in individuals with DMD in the early stages of the disease, assessed under different sensory conditions. The secondary aim was to verify whether COP velocity and displacement area increase in individuals at similar disease stages but under altered sensory conditions. 

## METHOD

 This cross-sectional, analytical study included individuals diagnosed with DMD, confirmed by muscle biopsy or genetic testing, who were receiving follow-up care at the Neuromuscular Diseases Investigation Sector of Universidade Federal de São Paulo (UNIFESP). 

 The inclusion criteria consisted of individuals classified in categories 2 (climb stairs using a handrail) and 3 (climbing upstairs with a handrail and slowly — taking more than 25 seconds to climb 8 steps) of the Vignos scale (disease staging), with a minimum age of six years. Vignos stages 2 and 3 were selected for comparison in this study because they are the two most functionally similar staging levels, differing only in the time required to complete the stair-climbing task. Additionally, individuals at these stages are able to maintain an upright position to perform the test. Given their movements patters and functional similarities, this study aimed to determine whether differences in postural control could already be present despite their comparable functional abilities. The Vignos scale consists of categories ranging from 1 to 10, with 1 indicating minimal clinical changes in posture and 10 representing a bedridden individual unable to perform functional activities.^8^ Exclusion criteria included individuals with non-union fractures; concurrent neurological diagnoses, or difficulty in understanding simple verbal commands. Additionally, participants with visual or auditory deficits that prevented assessment, a comfortable support base exceeding the equipment dimensions (45 x 26.5 cm), or an inability to maintain an orthostatic position within the equipment area for the required time (30 seconds) were excluded. Cognitive screening was conducted using the Mini-Mental State Examination (MMSE), which has a maximum score of 30 points. This scale was recommended by the Brazilian DMD consensus to assess cognitive performance.^5^ A minimum score of 10 points was required for participation, based on the study by Voos et al.,^9^ which reported that this is the lowest score for patients with DMD to understand simple verbal commands. Participants scoring below 10 were excluded. 

 The informed consent form was signed by the participants or their guardians upon agreeing to participate in the study. Children also signed an assent form. The study was approved by the Research Ethics Committee (*Comitê de Ética em Pesquisa* – CEP) of the University (CEP/UNIFESP) under the Certificate of Presentation of Ethical Appreciation (*Certificado de Apresentação de Apreciação Ética* – CAAE): 28327120.0.0000.5505 and report number 0112/2020. 

 In addition to measurements using a conventional force platform, recent studies, including a systematic review, have validate the use of the Wii Balance Board (WBB) for measuring postural control, highlighting its low acquisition cost and portability outside conventional laboratories.^ 10-13^ Thus, as a tool for assessing postural control, WBB (the Nintendo Wii force platform) was used, connected to a computer via Bluetooth. Participants stood in an orthostatic posture on the platform and were assessed with MSOT to identify the influence of sensory components on postural stability. Four sensory conditions were tested:

1. Maintenance of posture with eyes open and a stable base of support on the WBB, that is, without a balance pad;
2. Maintenance of posture with eyes closed and a stable base of support on the WBB, that is, without a balance pad;
3. Maintenance of posture with eyes open while standing on a balance pad (AIREX® foam type) placed over the WBB;
4. Maintenance of posture with eyes closed while standing on a balance pad (AIREX® foam type) placed over the WBB. Participants stood in static orthostatism on the WBB for 30 seconds in each condition.

MSOT is a suitable method to assess the contribution of proprioceptive, visual, and vestibular inputs to postural control.^14^

 Participants were assessed once by two physiotherapists who had been previously trained in the assessment tool and had over five years of clinical and research experience with the DMD population. Sample size calculation was performed using the *a priori* method (G*Power 3.1.7 software), with postural control variable values used as a reference. The statistical test “t-test: difference between two independent means” was applied, with power parameters of 0.80 and a significance level of 0.05. Reference values from the literature were used to determine the effect size, based on mean excursion area values of the COP in boys with DMD and a control group from the study by Futema and Sá,^15^ yielding an effect size of 2.0. Thus, the required sample size was 12 participants, with six in each group. Participants were invited to take part in the study based on their presence at the study site. Participants identified as Vignos stages 2 and 3 were invited to participate until the required sample was reached. There were no follow-up losses, as all participants completed the test properly, and none met the exclusion criteria. 

 Assessments were conducted with participants barefoot to eliminate any influence of footwear on measurements. The upper limbs were positioned along the body, and feet were placed at a comfortable width without exceeding shoulder width, as recommended in the literature for postural control assessment.^14^ In conditions requiring open eyes, a fixed visual target was placed on a wall 1 meter away at eye level, as recommended.^16^ In closed eyes conditions, participants were blindfolded. 

 The mean displacement velocity of the COP and the ellipse area (95%) formed by COP displacement were recorded using proper software. Clinical data were also collected, including weight, height, age, disease staging (Vignos Scale), use of lower limbs orthoses, frequency of physiotherapy sessions, if any, medication use, comorbidities, previous surgeries, and the presence of pain or dizziness. 

 Posturographic variables were collected and recorded using Brain Blox software,^17^ developed at the Neuromechanics Laboratory of the University of Colorado Boulder (USA). The recorded data were saved in Microsoft Excel (Redmond, CA, USA) format and analyzed using a second program, Clinical Posturography, which runs within Microsoft Excel. This second program was developed at the Bioengineering Laboratory of the Institute of Physical Medicine and Rehabilitation of Hospital de Clínicas of the School of Medicine of São Paulo (HCFMUSP) by a team of engineers and physiotherapists using Excel Visual Basic For Applications (VBA). This software was used to calculate the posturographic variables of interest: COP displacement velocity and displacement area. 

 Thus, the variables of interest were recorded for each level of disease staging (Vignos 2 and 3) in each MSOT subtest (4 sensory conditions). The ordinal categorical variable was disease staging (Vignos 2 or 3), and the continuous numeric variables were the postural control variables (COP displacement velocity and area). The analysis compared the mean COP displacement velocity and displacement area between the Vignos 2 and Vignos 3 groups in each sensory condition assessed. The analysis was carried out both intergroup and intragroup. The intergroup analysis compared the mean COP displacement velocity between the Vignos 2 and Vignos 3 groups in each sensory condition assessed. The intragroup analysis assessed whether differences existed in the mean COP displacement velocity within the same Vignos stage between the first and fourth sensory conditions — representing the simplest and most complex sensory conditions in the MSOT. 

 Shapiro Wilk’s test was applied to assess data normality, and Levene’s test was used to assess homogeneity. Given the normality of the data, independent samples Student’s t-tests were used to investigate the primary aim by comparing postural control variables between the Vignos 2 and Vignos 3 groups in each sensory condition. To investigate the secondary aim, dependent samples Student’s t-tests were used to compare postural control variables between sensory conditions 1 and 4. Statistical significance was set at p≤0.05. 

## RESULTS

 Six participants classified as Vignos 2 and six as Vignos 3 participated in the study, all of whom were boys with DMD. Table 1 shows the sample characteristics. Age ranged between 7 and 19 years old (mean 9.58, SD±3.22). Participants who did not use an orthosis to prevent or treat shortening of triceps surae musculature reported avoiding it due to discomfort, personal preference, perceived pain or limited encouragement from family members. The average weight of the sample indicates that the participants were, on average, at an appropriate weight range for their age. All participants were receiving corticosteroid therapy.

**Table 1:** Characterization of the boys diagnosed with Duchenne muscular dystrophy in Vignos stages 2 and 3.

	Age (years) (mean±SD)	Weight (kg) (mean±SD)	Receiving physical therapy (%)	Receiving corticosteroid therapy (%)	Uses AFO orthosis at night (%)
Vignos 2 group	9 (±1.9)	27.2 (±8.7)	100	100	50
Vignos 3 group	10.1 (±4.5)	35 (±14.0)	100	100	16.7

SD: standard deviation.

AFO: ankle foot orthosis.

Table 2 presents the results of the posturography test for each participant under each sensory condition. The posturographic variables analyzed were the mean COP displacement velocity and the ellipse area (95%) formed by the COP displacement during the assessment. The results indicate an increase in COP displacement velocity and COP displacement area with changes in MSOT evaluation condition. 

**Table 2:** Results of participants with Vignos 2 and 3 staging, in each sensory condition of the posturography test.

				Condition 1		Condition 2		Condition 3		Condition 4	
Vignos 2				COP-V(cm/s)	COP area(cm^2^)	COP-V(cm/s)	COP area(cm^2^)	COP-V(cm/s)	COP area(cm^2^)	COP-V(cm/s)	COP area(cm^2^)
Participant 1				2.2	4.6	1.4	17.0	3.8	10.9	4.3	14.4
Participant 2				1.7	4.1	1.7	6.1	2.1	5.8	3.7	10.8
Participant 3				2.7	9.0	3.9	11.6	4.0	15.7	5.0	18.4
Participant 4				1.8	2.3	1.9	5.5	2.1	5.1	3.8	10.6
Participant 5				4.0	10.2	3.8	15.4	4.3	20.9	5.9	29.5
Participant 6				1.9	3.9	1.9	4.3	4.6	10.5	5.6	12.5
Mean Vignos 2 (±SD)				2.4±0.9	5.7±2.2	2.4±1.1	9.9±5.5	3.5±1.1	11.5±6.0	4.7±0.9	16.0±7.2
Vignos 3											
Participant 7				1.6	2.5	1.9	5.9	3.9	6.1	3.0	17.8
Participant 8				4.2	8.1	4.9	9.6	5.7	23.9	6.7	28.5
Participant 9				2.5	4.1	3.7	9.5	4.8	7.3	6.7	18.9
Participant 10				3.0	20.6	3.2	17.3	1.7	34.5	4.1	23.9
Participant 11				2.4	5.5	3.1	9.7	3.9	6.9	5.2	12.6
Participant 12				3.6	2.4	3.8	9.6	4.9	10.5	5.4	13.7
Mean Vignos 3 (±SD)				2.9±0.9	7.2±6.9	3.4±1.0	9.7±4.0	4.2±1.4	14.9±7.7	5.2±1.4	19.2±6.0

Participant: study participant; V: displacement velocity; COP: center of pressure; SD: standard deviation.

Condition 1 being: eyes open and stable base of support without balance pad; condition 2: eyes closed and stable base of support without balance pad; condition 3: eyes open with balance pad; and condition 4: eyes closed with balance pad.

Table 3 compares participants in the Vignos 2 group with those in the Vignos 3 group. Statistical analysis shows that although all variables increased from Vignos 2 to Vignos 3, no significant differences were observed in any of the analyzed variable. 

**Table 3:** Results of the comparison between the Vignos 2 and 3 groups, for the center of pressure displacement velocity and center of pressure displacement area variables in each sensory condition of the modified sensory organization test.

	Vignos 2	Vignos 3	t-test	Effect size	Confidence interval-95% (lower to upper)
V-1 (cm/s)	2.4	2.9	p=0.352	-0.55	-1.70 to 0.61
V-2 (cm/s)	2.4	3.4	p=0.137	-0.95	-2.13 to 0.27
V-3 (cm/s)	3.5	4.2	p=0.371	-0.53	-1.68 to 0.63
V-4 (cm/s)	4.7	5.2	p=0.504	-0.38	-1.52 to 0.77
Area 1 (cm^2^)	5.7	7.2	p=0.638	-0.28	-1.41 to 0.86
Area 2 (cm^2^)	9.9	10.2	p=0.925	-0.06	-1.19 to 1.07
Area 3 (cm^2^)	11.5	14.9	p=0.547	-0.36	-1.50 to 0.78
Area 4 (cm^2^)	16.0	19.2	p=0.424	-0.48	-1.61 to 0.68

V-1: mean center of pressure displacement velocity in sensory condition 1 (eyes open and stable base of support without balance pad); V-2: mean center of pressure displacement velocity in sensory condition 2 (eyes closed and stable base of support without balance pad); V-3: mean center of pressure displacement velocity in sensory condition 3 (eyes open with balance pad); V-4: mean center of pressure displacement velocity in sensory condition 4 (eyes closed with balance pad); Area 1: center of pressure displacement area in sensory condition 1 (eyes open and stable base of support without balance pad); Area 2: center of pressure displacement area in sensory condition 2 (eyes closed and stable base of support without balance pad); Area 3: center of pressure displacement area in sensory condition 3 (eyes open with balance pad); Area 4: center of pressure displacement area in sensory condition 4 (eyes open and stable base of support without balance pad).

 Regarding the intragroup comparison, that is, examining results from sensory condition 1 *versus* sensory condition 4 of the MSOT within each Vignos stage, the findings were as follows: an increase in speed from 2.4 cm/s in condition 1 (eyes open, without the balance pad) to 4.7 cm/s in condition 4 (eyes closed, with the balance pad) for Vignos 2 (p=0.0012, effect size: -3.42; confidence interval: -5.60 to -1.21), and for Vignos 3, an increase in speed from 2.9 cm/s to 5.2 cm/s (p=0.0081, effect size: -2.03; confidence interval: -3.47 to -0.55). For COP displacement area across different sensory conditions, the Vignos 2 group showed an increase from 5.7 cm^2^ in sensory condition 1 (eyes open, without the balance pad) to 16.0 cm² in sensory condition 4 (eyes closed, with the balance pad) (p=0.0091, effect size: -2.26; confidence interval: -3.81 to -0.66). In the Vignos 3 group, displacement area increased from 7.2 cm^2^ in condition 1 to 19.2 cm ^2^ in condition 4 (p=0.0093, effect size: -1.95; confidence interval: -3.35 to -0.51). These results indicate a significant worsening in postural control performance with variations in sensory conditions in both groups. 

## DISCUSSION

 The present study aimed to determine whether there is an increase in COP velocity and displacement area in individuals with DMD at Vignos 2 and Vignos 3 stages and to assess whether these variables increase with changes in the sensory conditions of postural control assessment. Contrary to the initial hypothesis, no significant differences were observed. 

 The Vignos Scale^18^ evaluates overall muscular performance and functionality throughout disease progression. Individuals with DMD at the Vignos 2 stage can climb stairs only with the support of a handrail, while those at the Vignos 3 stage require handrail support and ascend slowly (taking more than 25 seconds to climb 8 steps).^19^ The decline in muscle strength is the primary factor contributing to reduce functionality in this population.^19^ The increased time required to perform functional tasks, as seen in the transition from Vignos 2 to 3 with slower stair climbing, reflects a decline in muscle strength and disease-related functional loss.^19,20^ To remain functional for as long as possible, individuals adopt compensatory movement patterns.^21^ The loss of strength observed in the progression from Vignos 2 to 3 leads individuals to use movement compensations during functional activities,^21^ such as increasing trunk range of motion and relying more on the upper limbs while climbing stairs. Muscle strength is a component of postural control, and despite its decline in the transition from Vignos 2 to 3, participants were still able to employ motor strategies to maintain postural control. It is possible that the decrease in strength was not yet substantial between these levels and that the sensory systems involved in postural control continued to function similarly in both stages. 

 Muscle strength is an important component for maintaining adequate postural control.^22^ In DMD, there is a progressive loss of muscle strength, which leads to postural deviations and functional deficits.^19,20,22^ As the disease progresses, muscle weakness and its secondary postural misalignments become insufficient to generate the stabilizing forces necessary to reestablish postural control.^22^ Additionally, the loss of muscle integrity over the course of the disease may impair the optimal functioning of muscle somatosensory receptors, neuromuscular spindles, and neurotendinous organs, thereby affecting somatosensory input for postural control regulation.^2^ Consequently, postural control deficits may occur throughout disease progression.^2,6,7^ The results of the present study, however, indicate no significant differences in postural control between the Vignos 2 and 3 groups. A numerical increase in COP displacement velocity and area was observed, but it was not statistically significant. Further studies involving later Vignos stages should be carried out to determine whether these values continue to increase with disease progression and, if so, at what point the decline in postural control becomes statistically significant. Since the participants in this study were still in the early stages of the disease, they may still be able to employ effective strategies to maintain postural control despite the onset of muscle weakness. 

 Regarding comparisons with the typical population, studies by Wolff et al.^23^ and Barozzi et al.^24^ identified that the average COP displacement velocity in typically developing children (without DMD) aged 7 to 18 years ranges from 1.45 to 0.87 cm/s in tests performed on a force platform in an upright position with eyes open and on a rigid surface, conditions similar to sensory condition 1 of the present study. When analyzing the present findings, participants with DMD already exhibit poorer postural control performance in the early stages of the disease, with COP displacement velocity values of 2.4 cm/s in Vignos 2 and 2.9 cm/s in Vignos 3. These results highlight the important of postural control assessment and early physical therapy intervention in this population. 

 In addition, consistent with the initial hypothesis, the results demonstrate a worsening in postural control within the same Vignos group. When sensory changes are introduced in the sensory changes organization test, participants’ postural control is further challenged. A study involving individuals with DMD in Vignos stages 1 to 3 also employed the MSOT and did not find an increase in the COP excursion area across different conditions. However, an increase in COP displacement velocity was observed when the test was performed with eyes closed and on a mobile platform.^2^ These results are partially consistent with those of the present study, in which both COP velocity and excursion area increased. An increase in velocity already indicates a decline in postural control with sensory changes in the test. However, participants in the study by Alvarez et al.^2^ may have still been able to implement strategies to stabilize their base of support, thereby minimizing changes in their COP excursion area. Additionally, there is a methodological difference between both studies. In the study by Alvarez et al.,^2^ a mobile platform was used to alter somatosensory input, whereas in the present study, a balance pad (AIREX® foam model) was used. The AIREX® foam may have contributed to greater changes in COP excursion area than a rigid moving surface. 

 The first MSOT condition is performed on an immobile, rigid surface with eyes open, whereas the fourth condition is performed on an unstable surface with eyes closed. Maintaining orthostasis requires constant integration of visual, vestibular, and somatosensory inputs.^3^ When sensory changes occur in a task or environment, a greater sensory challenge is introduced, as seen in MSOT. The transition from the first to the fourth condition reduces visual input, while somatosensory input becomes less precise due to the instability of the support surface.^25^ The worsening of postural control under these sensory conditions may be related to the muscle alterations present in individuals with DMD,^2^ resulting from both impairments in the effector system and deficits in somatosensory input. 

 Throughout the neurodegenerative progression, individuals may become more visually dependent for postural control.^2^ Eliminating visual input further impairs postural control, as visual proprioceptors assist in the recruitment and inhibition of different postural muscles.^26^ There is difficulty in processing information about postural orientation and verticality,^2^ as well as in muscle control needed to maintain adequate postural stability. Barrett et al.^3^ compared postural control performance between children with and without DMD. The DMD group exhibited worse performance, with higher COP displacement values than the control group (without DMD).^3^ In addition to the primary muscle deficit caused by the pathology, secondary deficits such as ankle, knee, and hip contractures require constant postural adaptations to maintain an upright posture.^3^ Children in the early stages of DMD showed values closer to those found in the control group. The authors suggest that this may be due to a lower prevalence of secondary deficits in individuals with DMD at earlier stages.^3^ Typical children require minimal muscle activity to maintain an upright posture, whereas children with DMD exhibited a more dynamic response with frequent activation of their remaining muscle fibers.^3^

 Maintaining effective postural control for as long as possible is essential for individuals with DMD, as adequate postural control is necessary for motor skills, performing daily activities, and preventing falls.^1,2^ In this study, no significant decline in postural control was observed between Vignos 2 and 3. Although no significant difference was found between these two stages, postural control performance in both groups is already considerable lower than that of individuals without DMD, highlighting the need for early interventions to preserve postural control performance for as long as possible before further deteriorations occurs.This study has certain limitations, such as the lack of longitudinal monitoring of disease progression, which could provide a better understanding of postural control alterations over time. In addition, only individuals in the initial Vignos stages were included. Future studies should assess individuals at other Vignos levels and incorporate longitudinal analyses. 

 The study of postural control has important clinical implications. It is essential to determine the point at which postural control begins to deteriorate significantly to enable early interventions aimed at improving balance, functionality, and reducing the risk of falls. Given the observed decline in postural control performance under different sensory conditions in individuals with DMD, early intervention strategies could include sensory system training, both in isolation and in tasks involving various sensory conflicts and challenges. By addressing both motor and sensory components in rehabilitation, it may be possible to optimize postural control outcomes. Understanding that postural control deteriorates under different sensory conditions highlights the importance of training sensory system function from an early stage of the disease. These systems can be trained either in isolation or in situations involving sensory conflicts during functional activities. However, no studies have yet investigated physiotherapeutic interventions specifically targeting these aspects to improve postural control in this population. Future interventional studies should explore this area of interest, as a rehabilitation program focused on early strength maintenance, deformity prevention, range of motion preservation, and postural alignment could be beneficial in sustaining postural control for as long as possible. In addition, such interventions may enhance postural control in situations involving sensory conflicts during daily tasks and environmental challenges. 

 In conclusion, no significant differences were found in postural control performance between children with DMD in Vignos stages 2 and 3. However, postural control significantly worsens with changes sensory conditions. 

## Data Availability

We declare that the database that originated the article is available upon request, with a corresponding author

## References

[1] Kim DH, An DH, Yoo WG (2018). Changes in trunk sway and impairment during sitting and standing in children with cerebral palsy. Technol Health Care.

[2] Alvarez MP, Fávero FM, Sá CS (2011). Avaliação do equilíbrio em pacientes com distrofia muscular de Duchenne. Acta Fisiatrica.

[3] Barrett R, Hyde SA, Scott OM, Dubowitz V (1988). Changes in center of gravity in boys with Duchenne muscular dystrophy. Muscle Nerve.

[4] Kyvelidou A, Stergiou N (2018). Visual and somatosensory contributions to infant sitting postural control. Somatosens Mot Res.

[5] Araujo AP, Nardes F, Fortes CP, Pereira JA, Rebel MF, Dias CM (2018). Brazilian consensus on Duchenne muscular dystrophy. Part 2: rehabilitation and systemic care. Arq Neuropsiquiatr.

[6] Sá CS, Fagundes IK, Araújo TB, Oliveira AS, Fávero FM (2016). The relevance of trunk evaluation in Duchenne muscular dystrophy: the segmental assessment of trunk control. Arq Neuropsiquiatr.

[7] D’Angelo MG, Berti M, Piccinini L, Romei M, Guglieri M, Bonato S (2009). Gait pattern in Duchenne muscular dystrophy. Gait Posture.

[8] Vignos PJ, Archibald KC (1960). Maintenance of ambulation in childhood muscular dystrophy. J Chron Dis.

[9] Voos M, Favero F, Dias K, Artilheiro M, Oliveira A, Caromano F (2015). Dissociation between motor and cognitive skills in subjects with Duchenne muscular dystrophy. Neuromuscul Disord.

[10] Clark RA, Mentiplay BF, Pua YH, Bower KJ (2018). Reliability and validity of the Wii Balance Board for assessment of standing balance: a systematic review. Gait Posture.

[11] Pérez-Alenda S, Carrasco JJ, Aguilar-Rodríguez M, Martínez-Gómez L, Querol-Giner M, Cuesta-Barriuso R (2017). Balance evaluation in haemophilic preadolescent patients using Nintendo Wii Balance Board®. Haemophilia.

[12] Holmes JD, Jenkins ME, Johnson AM, Hunt MA, Clark RA (2013). Validity of the Nintendo Wii® balance board for the assessment of standing balance in Parkinson’s disease. Clin Rehabil.

[13] Rohof B, Betsch M, Rath B, Tingart M, Quack V (2020). The Nintendo® Wii Fit Balance Board can be used as a portable and low-cost posturography system with good agreement compared to established systems. Eur J Med Res.

[14] Maranhão-Filho PA, Maranhão ET, Silva MM, Lima MA (2011). Rethinking the neurological examination I: static balance assessment. Arq Neuropsiquiatr.

[15] Futema E (2022). Limite de estabilidade e controle postural em meninos com Distrofia Muscular de Duchenne.

[16] Duarte M, Freitas SM (2010). Revisão sobre posturografia baseada em plataforma de força para avaliação do equilíbrio. Rev Brasil Fisioter.

[17] Cooper J, Siegfried K, Ahmed AA (2014). BrainBLoX: Brain and Biomechanics Lab in a Box Software (Version 1.0) [Software].

[18] Walton JN, Gardner-Medwin D (1974). Disorders of voluntary muscle.

[19] Allsop KG, Ziter FA (1981). Loss of strength and functional decline in Duchenne’s dystrophy. Arch Neurol.

[20] Goemans N, Wong B, Van den Hauwe M, Signorovitch J, Sajeev G, Cox D (2020). Prognostic factors for changes in the timed 4-stair climb in patients with Duchenne muscular dystrophy, and implications for measuring drug efficacy: a multi-institutional collaboration. PLoS One.

[21] Dazzi MA, Sá CS (2023). Gait and sit-to-stand motor compensation strategies in children and adolescents with Duchenne muscular dystrophy. Percept Mot Skills.

[22] Baptista CR, Costa AA, Pizzato TM, Souza FB, Mattiello-Sverzut AC (2014). Postural alignment in children with Duchenne muscular dystrophy and its relationship with balance. Braz J Phys Ther.

[23] Wolff DR, Rose J, Jones VK, Bloch DA, Oehlert JW, Gamble JG (1998). Postural balance measurements for children and adolescents. J Orthop Res.

[24] Barozzi S, Socci M, Soi D, Di Berardino F, Fabio G, Forti S (2014). Reliability of postural control measures in children and young adolescents. Eur Arch Otorhinolaryngol.

[25] Orendorz-Frączkowska K, Kubacka M (2019). The development of postural control in 6-17 old years healthy children. Part I postural control evaluation in modified Clinical Test for The Sensory Interaction on Balance in 6-17 old year children (mctsib). Otolaryngol Pol.

[26] Bronstein AM (2019). A conceptual model of the visual control of posture. Prog Brain Res.

